# Underlying Piezo2 Channelopathy-Induced Neural Switch of COVID-19 Infection

**DOI:** 10.3390/cells14151182

**Published:** 2025-07-31

**Authors:** Balázs Sonkodi

**Affiliations:** 1 Department of Health Sciences and Sport Medicine, Hungarian University of Sports Science, 1123 Budapest, Hungary; bsonkodi@gmail.com; 2 Department of Sports Medicine, Semmelweis University, 1122 Budapest, Hungary; 3 Faculty of Health Sciences, Institute of Physiotherapy and Sport Science, University of Pécs, 7624 Pécs, Hungary; 4 Physical Activity Research Group, Szentágothai Research Centre, 7624 Pécs, Hungary

**Keywords:** neuroCOVID, Piezo2 channelopathy, metabolic switch, microglia, glutamatergic synapse loss, autonomic dysregulation, ultradian rhythm

## Abstract

The focal “hot spot” neuropathologies in COVID-19 infection are revealing footprints of a hidden underlying collapse of a novel ultrafast ultradian Piezo2 signaling system within the nervous system. Paradoxically, the same initiating pathophysiology may underpin the systemic findings in COVID-19 infection, namely the multiorgan SARS-CoV-2 infection-induced vascular pathologies and brain–body-wide systemic pro-inflammatory signaling, depending on the concentration and exposure to infecting SARS-CoV-2 viruses. This common initiating microdamage is suggested to be the primary damage or the acquired channelopathy of the Piezo2 ion channel, leading to a principal gateway to pathophysiology. This Piezo2 channelopathy-induced neural switch could not only explain the initiation of disrupted cell–cell interactions, metabolic failure, microglial dysfunction, mitochondrial injury, glutamatergic synapse loss, inflammation and neurological states with the central involvement of the hippocampus and the medulla, but also the initiating pathophysiology without SARS-CoV-2 viral intracellular entry into neurons as well. Therefore, the impairment of the proposed Piezo2-induced quantum mechanical free-energy-stimulated ultrafast proton-coupled tunneling seems to be the principal and critical underlying COVID-19 infection-induced primary damage along the brain axes, depending on the loci of SARS-CoV-2 viral infection and intracellular entry. Moreover, this initiating Piezo2 channelopathy may also explain resultant autonomic dysregulation involving the medulla, hippocampus and heart rate regulation, not to mention sleep disturbance with altered rapid eye movement sleep and cognitive deficit in the short term, and even as a consequence of long COVID. The current opinion piece aims to promote future angles of science and research in order to further elucidate the not entirely known initiating pathophysiology of SARS-CoV-2 infection.

## 1. Introduction

The focal “hot spot” neuropathologies in COVID-19 infection [1] are revealing footprints of a hidden underlying collapse of a novel ultrafast ultradian Piezo signaling system within the nervous system. Paradoxically, the same initiating pathophysiology could underpin the systemic findings, namely the multiorgan SARS-CoV-2 infection-induced vascular pathologies and brain–body-wide systemic pro-inflammatory signaling, depending on concentration and exposure to infecting SARS-CoV-2 viruses [1]. This common initiating microdamage is suggested to be the primary damage or the acquired channelopathy of the Piezo2 ion channel, leading to a principle gateway to pathophysiology [2]. Primary damage, or the root cause of aging, has been long suspected in science [3]. An in-depth introduction of this pathophysiology initiating primary damage, or the loss of the ultrafast ultradian proton-based signaling, as being the acquired Piezo2 channelopathy could be found in a recent review [4]. Accordingly, several unexplained conditions and diseases are suggested to be initiated by acute, chronic or irreversible Piezo2 channelopathy, like delayed onset muscle soreness, hypertension, obesity, non-contact anterior cruciate ligament injury, dry eye disease, osteoporosis, psoriasis, amyotrophic lateral sclerosis, and even cancer, and many more, depending on the locus of the primary damage, genetic predisposition or environmental risk factors [4]. If this acquired Piezo2 channelopathy holds true, then it should have relevance in COVID-19 infection as well. Correspondingly, the current opinion piece introduces how Piezo2 channelopathy could explain the initiation of the observed disrupted cell–cell interactions, metabolic failure, microglial dysfunction, mitochondrial injury, glutamatergic synapse loss, inflammation and neurological states with the central involvement of the hippocampus and the medulla.

## 2. Proton Affinity Switch

It is considered that the main binding and entry loci for SARS-CoV-2 viruses are the angiotensin-converting enzyme 2 (ACE2), leading to the functional loss of ACE2 and its ectodomain shedding [5]. However, the neural entry of SARS-CoV-2 viruses is not always essential for COVID-19 neuropathology. In support, SARS-CoV-2 proteins could not often be detected inside brain neurons, but these proteins were rather situated in association with blood vessels [1]. It is noteworthy that a study on hamsters also showed a lack of neuroinvasion by SARS-CoV-2 viruses [6]. Correspondingly, the aforementioned shedding, like in the case of syndecans, has been emphasized to damage the membrane surface electrostatic micromilieu by losing negative charge [7]. Moreover, the SARS-CoV-2 spike-heparan sulfate (HS) interaction is needed for the “up/open” conformation for consecutive ACE interaction [8]. It is noteworthy that the Delta and Omicron variants’ spike protein mutations also enhance the positive charge in the likely HS binding site [9,10]. Glycosaminoglycan residues, such as HS side chains, are kosmotropic in nature according to the Hofmeister terminology [11], forming a strongly H-bonded network of water molecules and are suggested to give rise to conduction of protons released by the Piezo2 channels, hence explaining the ultrafast proton-based signaling initiation [12]. In addition, cell surface transmembrane HS proteoglycan syndecans are also binding sites for SARS-CoV-2 prior to syndecan-assisted cell entry [13,14]. This electrostatic micromilieu is important for Piezo2 function, since the ACE2 ectodomains and syndecans may be involved in proton transfer [7]. Therefore, a proton affinity switch and a resultant unidirectional proton reversal [15] should be considered as a consequence of SARS-CoV-2 binding to ACE2 and syndecans, and ACE2 ectodomains and syndecan shedding, leading the way to Piezo2 channelopathy. Indeed, electrostatic interactions are the primary factor that determines the binding affinity of the SARS-CoV-2 receptor binding domain (RBD) spike to ACE2 [16,17]. Since syndecans may be essential to Piezo2–Piezo1 crosstalk [4], the SARS-CoV-2 binding and shedding impairs this crosstalk, leading to impaired cell–cell interaction and cell orientation. As for the concrete biophysical mechanism for this non-synaptic Piezo2–Piezo1 crosstalk, the proton-conducting Grotthuss mechanism [18] was suggested [12]. The Grotthuss mechanism entails the concerted action of a large number of quasi-simultaneous proton hopping events along an H-bonded network of water molecules [18] that is suggested to be the interfacial water [4]. Hence, the current author proposes that SARS-CoV-2 binding to ACE2 and syndecans, and ACE2 ectodomain and syndecan shedding might be enough to alter the electrostatic micromilieu to a point of negative charge alteration to induce impaired proton transfer and a resultant proton affinity switch on Piezo2 function, leading to Piezo2 channelopathy and impaired Piezo2–Piezo1 crosstalk. Consequently, SARS-CoV-2 viral entry into neurons is not a must to induce Piezo2 channelopathy, explaining why viral proteins often could not be detected inside of brain neurons in neuropathology.

Berta et al. successfully showed the overlapping interaction site for the envelope (E) protein of SARS-CoV-2 and regulin [19]. Regulin is known to regulate sarco/endoplasmic reticulum (ER) calcium ATPases (SERCA). Hence, the E protein of SARS-CoV-2 acts like an exoregulin of SERCA2b, leading to the decreased SERCA-mediated ER calcium reload [19]. It is also known that the E protein of SARS-CoV-2 could construct homopentamers, leading to viroporins that permeate ions, like calcium. It is noteworthy that SERCA regulates the activation of the Piezo1 channel through direct protein–protein interaction [20]. Indeed, SERCA2 is shown to suppress Piezo1 [21]. However, it is proposed that the Piezo2 content of glutamatergic neurons is the critical locus for the primary damage in COVID-19 infection. In support, SERCA suppresses the mechanogating function of Piezo2 as well [22].

Accordingly, the SARS-CoV-2 RBD spike electrostatically binds to the ACE2 content of glutamatergic neurons, leading to shedding, but even more importantly, to viroporin formation. The E protein content may act like a suppressive exoregulin of SERCA that, in turn, disinhibits Piezo2. This is suggested to lead to the pathophysiology initiating over excessive stimulation of Piezo2, or to Piezo2 channelopathy [2]. This initiating overstimulation is in line with the increased aberrant level of oxidative phosphorylation (OXPHOS) under COVID-19 infection [23]. However, the binding of the SARS-CoV-2 RBD spike to ACE2 also inhibits neutral amino acid transport, including glutamine. Glutamine is heavily used by COVID-19, leading to glutamine depletion. This depletion disrupts the astrocyte neurite lactate shuttle (ANLS) as a result of Piezo2 channelopathy, leading to elevated glutamate, and, moreover, explains the VGLUT disconnection [1,2]. In parallel, the OXPHOS is depleted over time by the induced proton reversal, with the contribution of viroporin formation. From this point on, this proton reversal instigates the proposed neural switch, leading to excessive mitochondrial energy generation deficit and explaining mitochondrial injury.

Aside from ACE2 being an enzyme, it also carries the aforementioned role in the regulation of neutral amino acids, including glutamine [24]. Glutamine is critical for the replication of the SARS-CoV-2 virus, but for the host as well [25]. However, as SARS-CoV-2 binds to ACE2, it impedes glutamine uptake at critical loci of the host; therefore, it impairs glutaminolysis. It presents a locus minoris resistentiae, since glutamine and glutaminolysis are essential not only during rapid growth but also in pathological states like trauma, infection and sepsis [26]. In support, it is evident that COVID-19 infection depletes glutamine in a critical way [26]. As a result, elevated circulating glutamate, on the other hand, activates platelets [26]; moreover, excessive glutamate stimulation impairs ACE2 function even more through the aforementioned shedding [27].

Further consideration is the bi-compartmental mechanism and disruption of Piezo2 crosstalk of these types of microdamages [2]. Therefore, the neurovascular unit (NVU) of brain microdamage “hot spots” should be considered as bi-compartmental, where the neurons and their associated cells of the NVU should be considered as the focal one, and the endothelial cells of the affected blood vessels, the other systemic one. Endothelial Piezo2 content could be of utmost importance in this pathophysiology-initiating mechanism. Notably, syndecans, like syndecan-2 with endothel-specific expression, and ACE2 may also have a role in this phenomenon in the aforementioned fashion.

The proton affinity switch-derived proton reversal on Piezo2 is proposed to be a metabolic and energy generation switch on glutamatergic neuron terminals [2]. This reversed proton gradient may deplete OXPHOS-induced electron and proton motive force production to evolve on glutamatergic neuron terminals over time [2,28]. In support, SARS-CoV-2 highly inhibits OXPHOS downstream [29]. In addition, the proton influx might induce subthreshold calcium currents, but even more importantly, it may reverse electrons as well, leading to impaired mitochondrial energy generation by impairing proton-coupled electron transfer (mitochondrial injury), not to mention vesicular glutamate release [2]. This will be a metabolic switch because it could impair the aforementioned ANLS mechanism, leading to parallel lower energy-generating glutaminolysis in addition to glycolysis in the affected neuron terminals and compartments [2], but depletion of OXPHOS. These Piezo2-containing glutamatergic neuron terminals should be considered as metabolic pacer cells of the local micromilieu and in regard to NVUs; hence, not only astrocytes but also microglia are affected by this metabolic switch within these compartments. Furthermore, this metabolic switch is aggravated by the aforementioned SARS-CoV-2 infection-associated glutamine depletion. In support is the metabolic failure, mitochondrial injury and loss of glutamatergic terminals due to COVID-19 infection [1]. Additionally, Piezo2 channelopathy is proposed to lead to VGLUT disconnection, resultant switch/miswiring and glutamatergic synapse loss over time [15], as is also shown within the brain as a consequence of COVID-19 pathology [1].

Moreover, this proton reversal is also suggested to impair the aforementioned Piezo2–Piezo2 and Piezo2–Piezo1 crosstalk [2]. This disruption in the Piezo system is underpinned by the impaired concerted proton tunneling–electron tunneling, hence spoiling the quantum mechanical/molecular free energy stimulation capability of Piezo2-initiated ultrafast long-range proton and electron signaling fueled by OXPHOS [2].

## 3. Pro-Inflammatory Switch

It is important to note that the abovementioned impaired ANLS machinery-derived metabolic switch and the impaired Piezo2–Piezo1 crosstalk could activate astrocytes and microglia in a pro-inflammatory manner due to their local metabolic dependence on neurons. Microglia and astrocytes have anti-inflammatory responses when the NF-kappaB inflammatory signaling pathway is activated by reducing TNF-alpha and IL-6 due to Piezo1 activation [30]. Noticeably, Piezo2 channelopathy is suggested to induce TNF-alpha and IL-6, leading to NF-kappaB inflammatory signaling pathway activation [31]. However, the current author proposes that Piezo1-containing microglia and astrocytes cannot fulfill their anti-inflammatory activity due to the Piezo2 channelopathy-induced lost Piezo2-Piezo1 crosstalk within the given compartmental micromilieu, leading to pro-inflammatory activation. In addition, the metabolically neuron-independent Piezo1-containing macrophage activation could be dysregulated due to the Piezo2 channelopathy-induced impaired Piezo2–Piezo1 crosstalk as well [32]. In support, Piezo1 activation induces pro-inflammatory responses of macrophages, in contrast to anti-inflammatory responses of microglia and astrocytes [30]. However, SARS-CoV-2 viruses-induced Piezo2 channelopathy may skew this balanced mechanism towards a pro-inflammatory micromilieu, and that could be presented in the observed focal neuropathologies associated with neurovascular inflammation [1].

## 4. Hippocampus and the Medulla

The hippocampus is a prime site for adult neurogenesis in the brain. However, deceased SARS-CoV-2-infected patients exhibited loss of hippocampal neurogenesis [6]. This picture is accompanied by microglial activation and expression of IL-6 mostly in the hippocampus and the medulla oblongata [6]. An important finding is that neurogenesis and cognitive function are essentially dependent on Piezo1 of astrocytes [33]. A recent opinion piece highlighted the link between the positive effect of exercise on hippocampal neurogenesis and cognitive function with the involvement of Piezo1-containing platelets and the opposing negative effect of oxaliplatin on these territories [28]. Oxaliplatin is a platinum analog chemotherapy, and it is also suggested to induce a proton affinity switch on Piezo2 channels in an acute and chronic fashion that could explain the diminished hippocampal theta activity and neurogenesis, not to mention learning and memory deficits, coined as ‘chemobrain’ [28]. It is noteworthy that ‘chemobrain’ has very similar territories to ‘brain fog’, experienced as a result of COVID-19 infection. Furthermore, the aforementioned opinion piece stresses the importance of shear stress detecting Piezo1-containing liver sinusoidal endothelial cells and the Piezo2 channelopathy-derived impaired Piezo2–Piezo1 crosstalk as a locus minoris resistentiea under oxaliplatin treatment [28]. The caused hepatic sinusoidal damage, platelet activation and extravasation, not to mention spleen enlargement and thrombocytopenia, could also be analogous to COVID-19 infection and the finding of Fekete et al. [1]. Accordingly, Fekete et al. showed that viral RNA levels were similarly increased in the liver and the spleen, as could be observed in the brain [1]. Further in support, elevated IL-6 levels in the liver and the spleen showed a strong correlation with the IL-6 levels of the medulla, while increased IL-6 levels were also correlated with elevated microglial distribution heterogeneity in the dorsal medulla [1]. It is noteworthy that increased IL-6 points to the loci of Piezo2 channelopathy, as was earlier proposed [31]. In addition, autonomic imbalance was also suggested to be the direct result of Piezo2 channelopathy due to the impaired Piezo2–Piezo2 crosstalk of the compartmental microdamaged sites and the autonomous nervous system [2]. Hence, the higher involvement of the medulla under COVID-19 infection might be linked to the suggested Piezo2 channelopathies.

**Figure 1:**
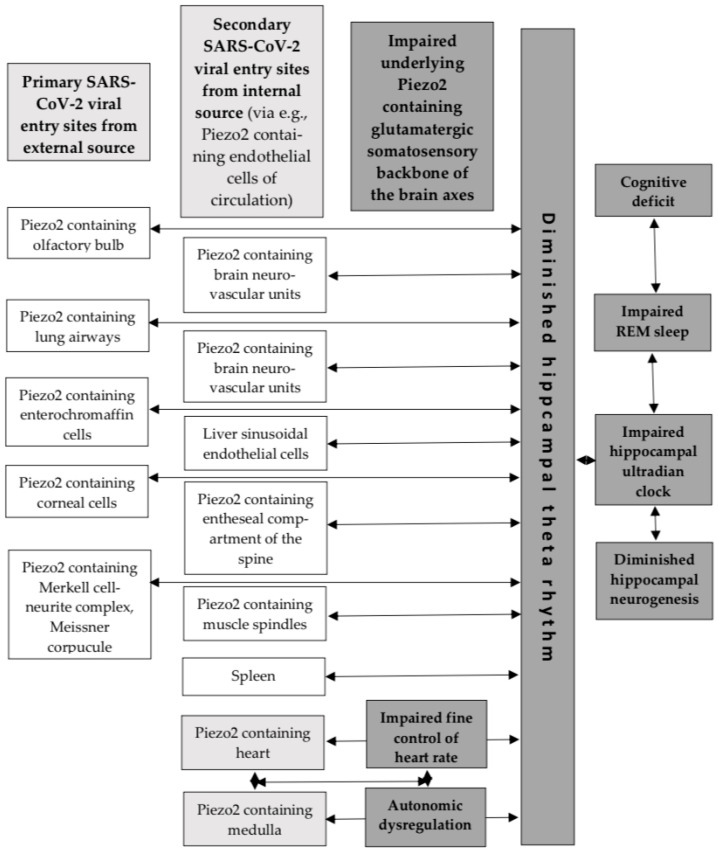
The novel ultrafast proton-based oscillatory synchronization mechanism to hippocampal theta rhythm, constructing the ultradian backbone of the brain axes. The dark gray boxes denote the COVID-19 infection-induced impairments and dysregulations.

Important indicative studies are noteworthy in support of the aforementioned functional link between the hippocampus and the medulla. It was theorized that Piezo2 synchronizes with hippocampal theta rhythm via an ultrafast proton-based synchronizational mechanism [4]. Earlier research showed that medullary neuron firing and hippocampal theta rhythm were phase-locked during periods of wakefulness [34]. The authors of this study even suggested that the hippocampal theta rhythm was involved in heart rate (HR) modulation [34]. Current research is also suggestive that stress-induced activation of Piezo2 participates in hippocampal fine control, or ultradian regulation, of HR [12]. Prior review introduced that voluntary movement, reflexes and autonomic control of HR are transiently related to hippocampal theta rhythm as part of ultradian brain rhythm [35]. Moreover, activated Piezo2-initiated underlying hippocampal ultradian synchronizational axes are suspected along the olfactory/prefrontal–hippocampal, muscle–brain, heart–brain, gut–brain, eye–brain, ear–brain, vestibular–brain and lung–brain axes, where the hippocampus is the temporary ultradian integratory hub, via hippocampal theta rhythm, not to mention constructing the hippocampal ultradian clock [36]. These underlying Piezo2-initiated ultradian axes are also theorized to be synchronized during rapid eye movement (REM) sleep via hippocampal theta rhythms as part of the hippocampal learning and memory consolidation process [4,36], as was shown earlier without implicating Piezo2 [34,35]. Correspondingly, the current author proposes that an underlying Piezo2-initiated proton-signaled synchronizational ultrafast ultradian medulla–hippocampal autonomic axis exists, in parallel to the analogous ultradian heart–hippocampal axis [12], for the fine control of HR. This is also in line with the aforementioned earlier research finding where medullary neuron firing and hippocampal theta rhythm were transiently phase-locked [34]. Remarkably, the Human Protein Atlas indeed shows Piezo2 content in the medulla (Figure 1).

The above-described COVID-19 infection-induced Piezo2 channelopathy not only impairs underlying ultradian synchronizational hippocampal axes along the muscle–brain, heart–brain, gut–brain, eye–brain, ear–brain, vestibular–brain and lung–brain axes depending on COVID-19 viral infection and intracellular entry, but also impairs autonomic regulation by impairing the Piezo2–Piezo2 crosstalk along the ultrafast ultradian heart–hippocampal and medulla–hippocampal autonomic axes. Moreover, COVID-19 infection initiates sleep disturbances [37]. The author of this manuscript suggests that these sleep disturbances are primarily due to the impairment of Piezo2-initiated synchronization along the aforementioned ultradian hippocampal axes, dysregulated REM sleep via hippocampal theta rhythms, and resultant hippocampal ultradian clock disruption; therefore, the hippocampal learning and memory consolidation process is also disturbed. Another important consideration on the long-term trajectory of SARS-CoV-2 infection is the frequent olfactory/prefrontal dysfunction-derived cognitive dysfunction, hyposmia and mental fatigue as a consequence of severe SARS in long COVID cases [38].

Finally, recent significant traumatic brain injury research shows the central role of Piezo2 in the stress-related defensive arousal response (DAR) [39]. DAR is a critical survival mechanism, turned on by perceived threat and evoked by visual and auditory inputs in relation to motor abilities [39]. It has been suggested that DAR and the underlying Piezo2 function are analogous to the proposed underlying Piezo2-initiated ultrafast ultradian hippocampal backbone of brain axes, including the proposed Piezo2-initiated medulla–hippocampus one, synchronized to hippocampal theta rhythm [36]. Accordingly, the author of this paper puts forward that acquired Piezo2 channelopathy impairs this ultrafast ultradian synchronization to the hippocampus as an underlying backbone of the brain axes due to SARS-CoV-2 infection, resulting in, e.g., autonomic dysregulation. This would explain the central involvement of the hippocampus and the medulla due to SARS-CoV-2 infection, as Fekete et al. demonstrated [1], and the findings of Pedemonte et al. that involved the temporary synchronization of the heart rate, medulla firing and the hippocampal theta rhythm under a homeostatic state [34], but not under SARS-CoV-2 infection.

## 5. Limitations

The author of this opinion piece explicitly highlights that the current paper is theoretical, reflects the opinion of the author, and is not yet validated experimentally from every aspect. However, it aims to promote future angles of science and research in order to further elucidate the not entirely known initiating pathophysiology of SARS-CoV-2 infection.

## 6. Conclusions

In summary, Piezo2 channelopathy should be considered as an underlying pathophysiology initiating primary damage, depending on COVID-19 viral infection and intracellular entry. Therefore, Piezo2-containing cells of the airways of the olfactory bulb, lung, enterochromaffin cells, cornea, endothelial cells of the circulation, muscle spindles, and entheseal compartments, not to mention certain brain areas, like the hippocampus and the medulla, should be considered as the pathophysiology initiating “hot spot” sites in the case of COVID-19 infection.

## Data Availability

No new data were created or analyzed in this study.

## Abbreviations

The following abbreviations are used in this manuscript:

ACE2	angiotensin-converting enzyme 2
ANLS	astrocyte neurite lactate shuttle
ER	endoplasmic reticulum
HS	heparan sulfate
NVU	neurovascular unit
OXPHOS	oxidative phosphorylation
RBD	receptor-binding domain
REM	rapid eye movement
SERCA	sarco/endoplasmic reticulum calcium ATPases
